# Factors related to postoperative vitreous hemorrhage after small-gauge vitrectomy in proliferative diabetic retinopathy patients

**DOI:** 10.1186/s12886-023-02940-2

**Published:** 2023-05-15

**Authors:** Meng Zhao, Aman Chandra, Jun Xu, Jipeng Li

**Affiliations:** 1 Department of Ophthalmology, Beijing Key Laboratory of Ophthalmology and Visual Science, Beijing Tongren Hospital, Beijing Tongren Eye Center, Capital Medical University, No1. Dongjiaominxiang street, Dongcheng District, Beijing, 100730 China; 2 Mid & South Essex NHS Foundation Trust (Southend University Hospital) Prittlewell Chase Essex SS00RY, Anglia Ruskin University, Cambridge, UK

**Keywords:** Perioperative management, Proliferative diabetic retinopathy, Pars plana vitrectomy, Postoperative vitreous hemorrhage, Anticoagulation, Antiplatelet

## Abstract

**Background:**

The role of anticoagulation or antiplatelet on post-vitrectomy vitreous hemorrhage (POVH) in patients with proliferative diabetic retinopathy (PDR) is rarely investigated in the small-gauge vitrectomy era. We investigate the relationship between the long-term use of those medications and POVH in a group of PDR patients.

**Methods:**

A retrospective cohort study was carried out in a group of PDR patients who underwent small-gauge vitrectomy in our center. The baseline data on diabetes, diabetic complications, long-term use of anticoagulants and antiplatelet agents, ocular findings, and vitrectomy details were collected. The occurrence of POVH was recorded during at least three-month follow-up. Factors related to POVH were analyzed using logistic analysis.

**Results:**

During a median follow-up of 16 weeks, 5% (11/220) of patients had POVH, and 75 had received antiplatelet or anticoagulation agents before the operation. Factors related to persistent POVH were the use of antiplatelet or anticoagulation agents (5.98, 1.75–20.45, p = 0.004), the presence of myocardial revascularization (130.65, 3.53-4834.50, p = 0.008), the presence of coronary artery disease (CAD) treated with medicine (56.52, 1.99–1604.06, p = 0.018), and younger age (0.86, 0.77–0.96, p = 0.012). For those receiving preoperative antiplatelet or anticoagulation agents, the probability of developing POVH was higher in the patients whose previous therapy was adjusted compared to those with continued therapy (p = 0.02 by Log-rank test).

**Conclusions:**

We identified long-term use of anticoagulation or antiplatelet medication, the presence of CAD, and younger age as three independent factors related to POVH. In PDR patients on long-term antiplatelet or anticoagulation medications, particular attention should be given to controlling intraoperative bleeding, and follow-up for POVH should be scheduled.

## Background

Proliferative diabetic retinopathy (PDR) is the leading cause of blindness in working-age individuals [1]. Pars plana vitrectomy (PPV) may be required to treat the complications of PDR, such as vitreous hemorrhage (VH) and tractional retinal detachment (TRD), to prevent patients from severe, irreversible vision loss. Postoperative vitreous hemorrhage (POVH) is a common PPV complication and occasionally requires reoperation.

PDR, as a complication of diabetes, is often combined with diabetic cardio-cerebral vascular diseases (CVDs) or chronic kidney diseases (CKDs) [2–7]. Systemic diabetic complications may require long-term anticoagulation or antiplatelet therapy to prevent thromboembolism. The surgeon often must balance the risk of thromboembolism and the risk of bleeding in the perioperative adjustment of anticoagulation and antiplatelet therapy. The perioperative guidelines on the adjustment of anticoagulation and antiplatelet therapy in noncardiac surgery show that in acute coronary syndrome, for patients with percutaneous intervention or drug-eluting stents, aspirin is recommended to be continued and clopidogrel should be restarted as soon as possible after surgery [5]; in patients with atrial fibrillation a mechanical heart valve, or venous thromboembolism, warfarin is typically stopped five days before surgery and resumed within 24 h postprocedure in cases of moderate bleeding risk, and low-molecular-weight heparin (LMWH) bridging therapy may be considered in the perioperative period [8]; in patients with CKD, heparin is recommended. The LMWH bridging therapy is commonly used in dialysis patients or diabetic CKD patients during the perioperative period [3]. There is no guideline or consensus on the perioperative adjustment of anticoagulation or antiplatelet therapy in diabetic vitrectomy. The consensus regarding ocular surgery recommends that there is no need to stop anticoagulation therapy in only cataract surgery, which is regarded as a procedure with a minimal bleeding risk [9].

In the 20-gauge vitrectomy era, without intravitreal anti-vascular endothelial growth factor (IV anti-VEGF) agents, endodiathermy, or intraoperative photocoagulation, the POVH occurrence in diabetic vitrectomy was relatively high, 39–63% [10, 11], and was related to the continued use of antiplatelet or anticoagulant agents therapy during vitrectomy [12]. However, in the small-gauge vitrectomy era, with the use of IV anti-VEGF agents, intraoperative photocoagulation, and endodiathermy, POVH occurrence has decreased dramatically to 3.1–18.9% [13–17]. It is believed to be unrelated to the continued use of anticoagulants in dialysis patients [18] or antiplatelet therapy [12, 14, 18, 19]. In addition, a survey on the consideration of discontinuing anticoagulation or antiplatelet therapies during vitrectomy shows that only 9% of surgeons feel that they would stop anticoagulation if possible for diabetic vitrectomy and retinectomy [20].

Extensive vitreoretinal adhesion and dissection of the fibrovascular proliferative membrane (FVM) are known risk factors for POVH [13, 17, 21, 22]. Additionally, the previous studies mentioned above did not deal with patients with extensive FVM cases, and data about the relationship of POVH to anticoagulation and antiplatelet therapies in patients with severe PDR in the small-gauge vitrectomy era are limited. Thus, we examined a series of PDR patients, including those with severe cases with extensive FVM and TRD, investigated the relationship of POVH to anticoagulation and antiplatelet therapies, and sought to address these research gaps [21, 22].

## Methods

This retrospective study included a cohort study of consecutive hospitalized patients with PDR who underwent PPV at our hospital during 1.1.2016–12.31.2017. If there was bilateral involvement, the eye with more severe ocular manifestations was selected. This study was approved by the Ethics Committee of Beijing Tongren Hospital, and it adhered to the tenets of the Declaration of Helsinki. The requirement for informed consent was waived.

The inclusion criteria were as follows: (1) PDR patients with TRD or VH who were hospitalized for PPV, (2) patients with available medical and operative documentation and laboratory tests to confirm diabetic systemic complications; (3) patients with details on the perioperative adjustments of anticoagulation and antiplatelet therapies; (4) patients with at least a three-month follow-up record. Exclusion criteria included: (1) VH which was found to be unrelated to PDR during PPV; (2) PDR eyes that underwent PPV for an epiretinal membrane or macular hole; (3) eyes with a history of PPV; (4) a lack of information on the history of DM, hypertension (HTN), evaluation on DM-related CVD, or CKD; (5) a lack of information on the use of anticoagulation, antiplatelet medication, or operative records; or (6) a lack of at least 3-month follow-up data.

Demographics, including age; gender; preoperative characteristics, including DM duration and DM medication, and the history of diabetes-related complications, including stroke [23] coronary artery disease (CAD) [23], congenital heart failure, diabetic foot and CKD [24], [25], and HTN [26]; anticoagulation or antiplatelet medications; and ocular characteristics, including the history of previous photocoagulation, cataract phacoemulsification extraction, IV anti-VEGF agents, visual symptom duration, visual acuity (VA), lens status, and the presence of iris neovascularization and neovascular glaucoma, were reviewed from the patients’ charts.

All the patients included in our study underwent preoperative anesthetic assessment. Physicians treated each patient with abnormal systemic conditions detected during preoperative screening. After the surgeon and the physician balanced the systemic risk of stopping anticoagulation or antiplatelet therapy and the benefit of PPV, the previous therapy was adjusted or left unchanged. The adjustment or maintenance of antiplatelet or anticoagulation medications in the perioperative period was recorded. The surgeon confirmed the restart of previous anticoagulation or antiplatelet therapy after PPV based on the post-PPV ocular signs for active bleeding. Adjustments to the renal dialysis plans and antiplatelet or anticoagulation therapy were recorded. PPV was postponed in patients who had had percutaneous transluminal coronary angioplasty, coronary artery bypass grafting, or cerebrovascular event in recent six months, especially those who were using dual antiplatelet therapy or had drug-eluting stent implants.

The patients had retrobulbar anesthesia or general anesthesia. All patients underwent 23/25 gauge 3-port PPV (Constellation Vision System, Alcon, Fort Worth, Texas; USA) (Stellaris PC, Bausch & Lomb, USA) with 5000 cuts/min vitreous cutting rates. Patients with active fibrovascular epiretinal proliferative membranes (FVMs) or severe VH obscured the fundus were treated with IV ranibizumab (IVR) within seven days before PPV. Pre-PPV IVR was recorded.

After a core vitrectomy, the extent of the FVM, the presence of macular-involving TRD, and photocoagulation scars were recorded.

Triamcinolone acetonide-assisted PPV was performed. Anterior-posterior vitreoretinal traction was released as much as possible. The induction of posterior vitreous detachment, FVM dissection, segmentation, delamination, endodiathermy, and the drainage of subretinal fluid, retinectomy, and intraocular tamponade was performed according to each patient’s particular need. Peripheral vitrectomy with scleral indentation and endolaser photocoagulation was performed for each patient. Intraocular pressure elevation, perfluorocarbon liquids, and endodiathermy were used to handle the intraoperative bleeding.

The severity of intraoperative bleeding was recorded as follows:

grade 0: none;

grade 1: minor bleeding stopping spontaneously or with transient bottle/pressure elevation;

grade 2: moderate to severe bleeding requiring endodiathermy or with the formation of broad sheets of clots [27].

Incomplete scatter photocoagulation was defined as a lack of preexisting photocoagulation in all 4 quadrants or needing more than 500 laser spots during PPV. The operation time was calculated from when the first PPV trocar incision was made to removing the eyelid speculum after PPV. The use of additional procedures, including cataract extraction, silicone oil tamponade, photocoagulation, and endodiathermy was recorded. The laser points were recorded.

The extension of FVM was recorded as follows:

grade 0: the absence of any adhesion;

grade 1: multiple point adhesions with or without one broad adhesion (broad adhesion was defined as focal adhesion at three sites or more);

grade 2: 1–3 broad adhesions posterior to the equator;

grade 3: 3 + broad adhesions posterior to the equator or 2 or fewer in-quadrant with adhesions anterior to the equator;

grade 4: broad adhesions anterior to the equator at multiple sites [28, 29].

FVMs were classified as predominantly neovascular, mixed neovascular and fibrotic, and predominantly fibrotic [27].

### Follow-up

All patients were followed for at least three months on a monthly schedule. The follow-up period was recorded as the time to develop POVH in patients who developed POVH. The patients with POVH were followed on a weekly schedule. Spontaneously resolved POVH was defined as the vitreous hemorrhage resolved without surgical intervention within six weeks of follow-up. Severe persistent POVH was defined as non-clearing vitreous hemorrhage needing a second PPV. The reoperation was carried out in the following conditions:1) severe persistent POVH in patients without silicone oil tamponade for more than six weeks without secondary glaucoma; 2) severe persistent POVH caused ghost cell glaucoma;3) persistent POVH under silicone oil tamponade was recorded when the patients had newly developed active bleeding around the disc or progressive clot formation that caused shallowing of the anterior chamber and elevation of intraocular pressure. The incidence of persistent POVH and ocular findings for POVH was also recorded.

### Statistical analysis

Statistical analysis was performed using R version 3.20 (http://www.R-project.org). Patient characteristics were retrieved from their medical charts and recorded in Epidata Entry Client version 2.0.3.15 (http://epidata.dk). The corrected VA results were converted to logMAR values for statistical analysis. Means and standard deviations (SDs) were calculated for continuous variables with a normal distribution. Medians with quartiles were calculated for continuous variables with a nonnormal distribution. The t test or Mann–Whitney U test was used for continuous variables. The chi-square test or Fisher’s exact test was used for discrete variables.

To investigate the impact of systemic complications and ocular characteristics on POVH, PDR patients were divided into patients with POVH and patients without POVH. The baseline systemic condition and medications, ocular findings, silicone oil tamponade, and intraoperative bleeding were compared between the two groups. Variables with a p value less than 0.3 were further enrolled in a binary backward stepwise logistic regression model. Each time, one variable was included or excluded from the model by comparing the Akaike Information Criterion (AIC) value; the model with the lowest AIC was chosen. Final p value for significance was set at 0.05.

To investigate the difference in the time to development of POVH, the survival analysis with the Kaplan-Meier survival curve was carried out in patients under long-term anticoagulant or antiplatelet medicines. The Log-rank test was carried out on POVH between patients whose previous therapy was adjusted or continued.

## Results

### Incidence of persistent POVH

During a median follow-up of 16 weeks, ranging from 1 to 26 weeks, 33 out of 220 (15.0%) patients had POVH. Among them, 11 out of 220 patients (5.0%) had a severe persistent POVH and underwent reoperations. The median time from the initial PPV to the repeated PPV was 9 days, (range from 1 to 35 days). In addition, 22 (10.0%) eyes had a spontaneously resolved VH and were not included in further analyses. The site of bleeding that caused severe persistent POVH was found during the reoperation as the margin of retinectomy (3), residual FVM on disc (3), residual FVM on retina elsewhere (2), residual vitreous cortex (2), unknown (1).

### Investigation of indications for antiplatelet and anticoagulation therapy

The abnormal systemic conditions requiring antiplatelet or anticoagulation therapy were identified as CKD, CVD, atrial fibrillation, HTN, and diabetic foot (n = 141). Among the 141 patients requiring antiplatelet or anticoagulation therapy, 66 patients did not receive any medication due to an unawareness of their abnormal systemic conditions; 13 patients discontinued their treatment after the onset of ocular symptoms; and 34 patients were on aspirin, five on heparin, one on dabigatran, 15 on dual antiplatelet therapy (aspirin and clopidogrel or dabigatran), and seven on combined therapy (Table 1).

**Table 1:** The use of anticoagulation and antiplatelet medications in PDR patients at presentation

Patients with systemic abnormalities required anticoagulation or antiplatelet medications	number
without any medication	66
discontinued the medications after onset of ocular symptoms	13
aspirin monotherapy	34
heparin	5
dabigatran	1
dual antiplatelet therapy	15
antiplatelet combined with anticoagulation medications	7

**Fig. 1:**
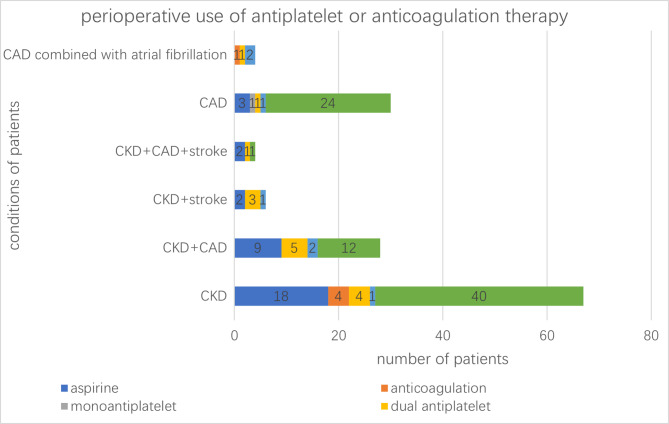
The number of patients with varied systemic conditions taking antiplatelet or anticoagulation medications. More complex therapy was used in patients with more severe systemic conditions. CAD: coronary artery disease; HBP: hypertension; CKD: chronic kidney disease

The reasons for combined therapy were CKD combined with HTN (5/67), CKD combined with CAD and HTN (7/28), CKD combined with stroke and HTN (4/6), CKD combined with CAD and stroke (1/4), CAD (2/30), and CAD with AF (3/4). There were 109 patients with stage 2 or more severe CKD; there were 36 patients on anticoagulation or antiplatelet therapy and 5 out of 12 dialysis patients on heparin. There were 66 patients with a diagnosis of CAD. Among them, there were 13 patients on antiplatelet and/or anticoagulation therapy (Fig. 1).

In patients on aspirin monotherapy, aspirin was discontinued for at least one week before surgery in 27 patients (79.4%). Clopidogrel (9/15) was discontinued for at least five days before surgery in patients with CAD treated with dual antiplatelet therapy. In patients with dual antiplatelet therapy, aspirin was continued, while dabigatran (1) and clopidogrel (6/15) were discontinued. Warfarin (7/7) was stopped and bridged with low-weight-molecular heparin three days before the operation. In patients on dialysis, heparin (5) was stopped and bridged with LMWH during the perioperative period. There were 22 patients (35.5%) whose antiplatelet or anticoagulation therapy was not changed due to systemic abnormalities, and none of them developed POVH.

All anticoagulation and antiplatelet therapies were restarted within one week after PPV when the absence of rebleeding in the fundus was confirmed.

### Investigation of operative ocular findings

One hundred ninety patients (86.4%) had at least one dose of an IV anti-VEGF agent before PPV. Thirteen patients (5.91%) received combined phacoemulsification cataract extraction. Sixty-five patients (29.5%) had retinectomy for drainage of subretinal fluid or separation of the FVM in the periphery. Ninety-eight patients (44.5%) had grade 3–4 FVM. Sixty-nine patients (31.4%) had macular-involved TRD. Eighty-eight patients (40.0%) had grade 2 intraoperative bleeding for which endodiathermy was needed. The median laser points were 1044 (791), and 181 patients (82.3%) had incomplete PRP requiring more than 500 laser spots during PPV. Seventy-four patients (33.6%) had silicone oil tamponade.

### Factors related to the occurrence of severe persistent POVH

Compared to the patients without persistent POVH, patients with persistent POVH were younger (median (IQR), 45.4 (9.3), 52.9 (17.1)y, p = 0.03), and had a higher proportion of patients on dialysis (3/11, 9/209, p = 0.01) and a higher proportion of patients who had combined CAD (11/11, 57/209, p < 0.001). More patients with persistent POVH than patients without POVH were on dual antiplatelet therapy (4/11, 11/209, p < 0.001) and dual antiplatelet combined with anticoagulation therapy (3/11, 4/209, p < 0.001). However, the two groups did not significantly differ in the ocular findings and PPV details (Table 2).

**Table 2:** The characteristics between patients with persistent postoperative vitreous hemorrhage and those without

	Univariate Analysis			Logistic regression for multiple analysis		
	Patients without POVH (209)	Patients with POVH (11)	P value	Odd ratio	95%CI	P value
age at presentation (y, median(IQR)	45.4 (9.3)	52.9 (17.1)	0.03	0.86	0.77–0.97	0.01*
Gender (male,n,%)	118, 56.4%	8, 72.7%	0.36			
duration of DM (y, median(IQR)	120 (132)	196 (102)	0.12	1.01	0.99–1.02	0.051
HTN (n,%)	105, 50.2%	8, 72.7%	0.25	1.59	0.19–13.10	0.67
CAD (n,%)			< 0.001			
received coronary revascularization surgery	10, 4.8%	4, 36/3%	< 0.001	130.65	3.53–4834.50	0.008*
with only medical treatment	47, 22.4%	6, 54.5%	0.04	56.52	1.99–1604.06	0.018*
Stroke (n,%)	15, 7.2%	1, 9.1%	0.99			
Impaired renal function (n,%)	58, 27.8%	1, 9.1%	0.29			
under dialysis (n)	9	3	0.01	8.72	0.46-167.01	0.15
under dialysis using heparin (n)	5	3	0.99			
antiplatelet or anticoagulation therapy (n,%)						
aspirin	31, 14.8%	3, 36.4%	0.49	5.98	1.75–20.45	0.004*
dual antiplatelet	11	4	< 0.001			
dual antiplatelet combined anticoagulation	3	4	< 0.001			
ocular findings (n,%)						
VA < 0.02 (n,%)	121, 57.9%	6, 54.5%	0.99			
incomplete PRP or without PRP (n,%)	171, 81.8%	10, 90.9%	0.69			
preoperative IVR	180, 86.1%	10, 90.9%	0.99			
operation time (min, median(IQR)	90 (60)	100 (40)	0.41			
TRD involving macular	65, 31.1%	4, 36.3%	0.74			
dense VH obscure the detail of fundus	92, 44.4%	6, 54.4%	0.55			
grade 3 FVM	94, 45.0%	4, 36.3%	0.76			
retinectomy	60, 28.7%	5, 45.4%	0.31			
grade 2 intraoperative bleeding	85, 40.7%	3, 27.3%	0.53			
silicone oil tamponade	70, 33.5%	4, 36.3%	0.99			
laser points (median(IQR))	1040 (801)	1431 (845)	0.37			

DM: diabetes milieus; VH: vitreous hemorrhage; CKD: chronic kidney disease; VA: visual acuity; PRP: pan-retinal photocoagulation; IVR: intravitreal injection of ranibizumab; FVM: fibrovascular proliferative membrane. *: p value <0.05.

Logistic regression showed that factors related to persistent POVH were the use of antiplatelet or anticoagulation agents. The risk of POVH was increased 5.98 (1.75–20.45, p = 0.004) times when one or more antiplatelet or anticoagulation therapy was added to aspirin monotherapy, the presence of CAD was treated by CABG or PTCA (130.65, 3.53–4834.50, p = 0.008), or the presence of CAD was treated with medical treatment (56.52, 1.99–1604.06, p = 0.018), and younger age (0.86, 0.77–0.96, p = 0.012). Dialysis with heparin was not a related factor (p = 0.15) (AIC = 48.45, AUC = 97.9) (Table 2).

We further investigated antiplatelet or anticoagulation therapy in patients with persistent POVH (10/11). The underlying diseases that required antiplatelet or anticoagulation therapy were myocardial infarction (6), dialysis with CAD (1), CAD combined with stroke (1), dialysis without CVD (1), and deep venous thrombosis (1). The POVH developed in all cases after they restarted the previous anticoagulation or antiplatelet therapy .

**Fig. 2:**
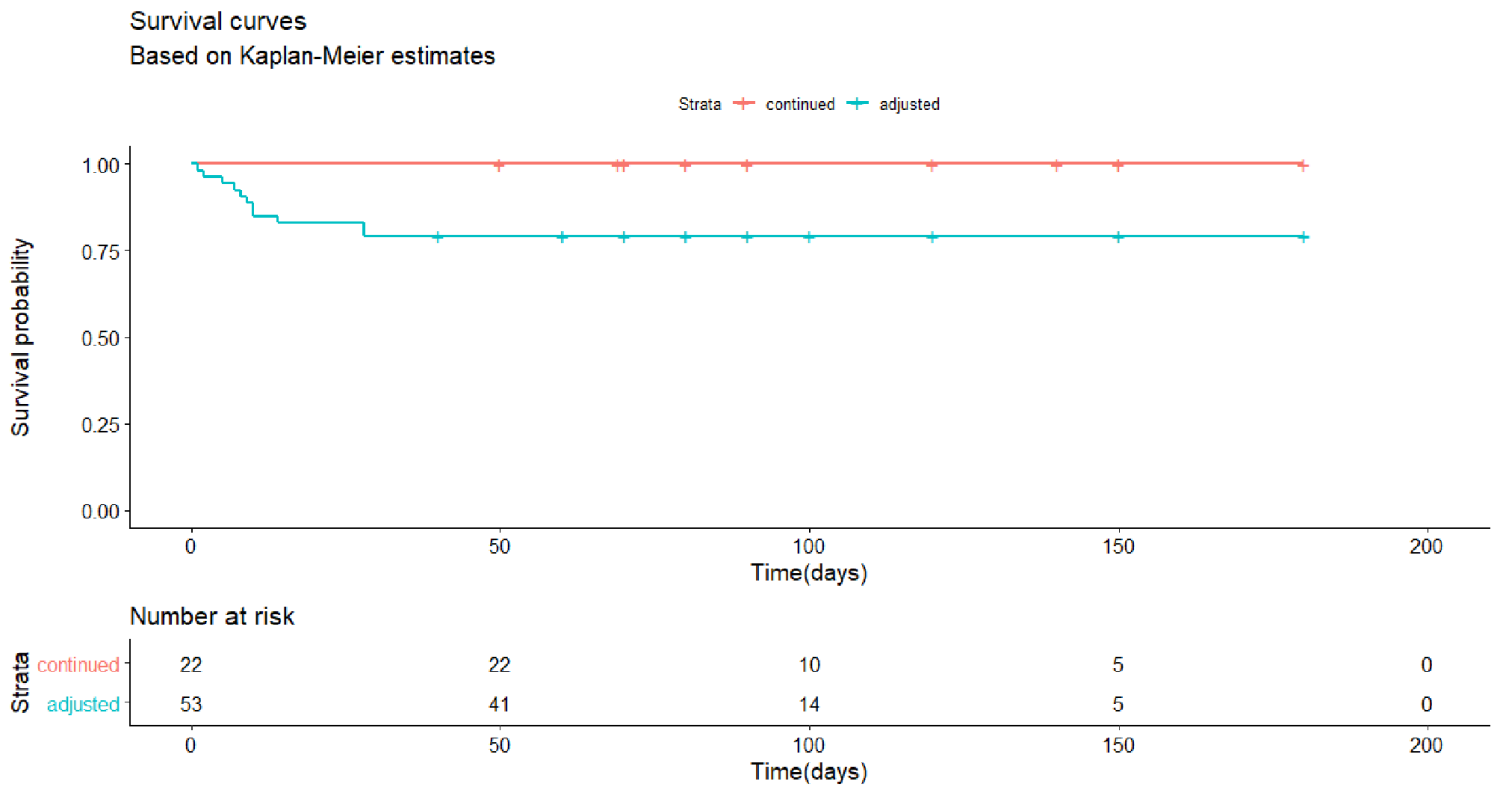
The survival curve of the development of POVH between patients whose anticoagulant antiplatelet therapy was adjusted or continued in a group of PDR patients receiving preoperative antiplatelet or anticoagulation agents

We only performed survival analysis on POVH in patients receiving preoperative antiplatelet or anticoagulation agents. A higher proportion of patients developed POVH in patients whose previous anticoagulant or antiplatelet therapy had been adjusted before PPV than in patients whose previous therapy was continued (11/53, 0/22, p = 0.01). In addition, the log-rank analysis showed that the probability of developing POVH was higher in the patients with adjusted therapy compared to those with continued previous therapy (p = 0.02, Fig. 2).

## Discussion

The current study found that 33 out of 220 (15.0%) patients had POVH; the long-term use of anticoagulation and antiplatelet medication in combination with aspirin use was positively related to POVH, which was not affected by the perioperative adjustment of the therapies. Our data supported the previous findings that adjusted or continued anticoagulation and antiplatelet therapy were unrelated to the occurrence of POVH in diabetic vitrectomy. Moreover, our data added that the lack of a correlation between continued anticoagulation and antiplatelet therapy and POVH was independent of the severity of PDR. Surprisingly, the presence of CAD was related to POVH in our study.

Heterogeneity exists among studies regarding patient characteristics, which means that the POVH from one investigated group could not be compared with others. The divergence of the occurrence of POVH may be affected by vitrectomy management, systemic conditions, FVM characteristics, TRD, IVR, PRP, and anticoagulation or antiplatelet therapy. Our studied population differed from those of other studies.

Our cohort of PDR patients had a high prevalence of CKD, CAD (141/220), and (CVD (141/220). Antiplatelet use is recommended in DM patients to reduce CVD mortality risk [30]. The long-term use of combined antiplatelet or anticoagulation is required in most diabetic patients with CKD or CVD [2–7], especially in patients with AF or deep vein thrombosis [7]. Contrary to expectations, 66 out of 141 patients with CKD or CVD were not on proper anticoagulation or antiplatelet therapy due to their unawareness of the combined systemic conditions prior to preoperative assessment.

Most patients were instructed to discontinue anticoagulation, antiplatelet medications, or bridging therapy with LMWH before PPV (64.5%). Aspirin monotherapy was discontinued for at least one week (27/34), dual antiplatelet therapy was switched to aspirin monotherapy for at least five days (15/15), and anticoagulation therapy was replaced with warfarin (7), and heparin (5) was bridged with LMWH during the perioperative period. The anticoagulation or antiplatelet plans of twenty-two patients (35.5%) were not changed due to systemic abnormalities. Thirteen patients stopped anticoagulation medication after the onset of ocular symptoms without physician consultation. To reduce the risk of thrombosis, most patients’ previous anticoagulation or antiplatelet medications were restarted one to two days after PPV and following post-PPV ocular evaluation of the possibility of active bleeding, according to the American College of Chest Physicians guidelines for the perioperative management of anticoagulation and antiplatelet therapy [31]. Our data demonstrated that this practice of altering the systemic therapy in advance of PPV surgery (53) might increase the occurrence of POVH compared to a small sample of patients who continued the previous therapy (22). It should be explored in a further study enrolling a larger sample of PDR patients with adjusted or continued anticoagulant or antiplatelet therapy.

44.5% of patients had disc-involved FVMs, 31.4% of patients had macular-involved TRDs, 29.5% had retinectomy, 40.0% had severe intraocular bleeding requiring endodiathary, 82.3% of patients had incomplete PRP, and 33.6% of patients with silicone oil tamponade. Previous studies have shown that retinectomy 7, [19], residual epiretinal neovascularization [15, 21], disc-involved FVM [13], and incomplete PRP [14] are risk factors for POVH development. We had a high percentage of complicated cases with a high risk of POVH.

Persistent POVH can significantly hinder visual rehabilitation and may require additional PPV. We found that 33 out of 220 (15.0%) patients had POVH. Among them, 11 out of 220 (5.0%) eyes had a severe persistent VH and underwent reoperations. Novak first reported the rate of the occurrence of POVH in 20-gauge diabetic vitrectomy as 63% on a postoperative day one and the reoperation rate as 9% in 1984 [11]. The occurrence of POVH has dropped to 3.1–18.9% since the development of new vitrectomy instruments and techniques and IV-anti VEGF [13–17]. Our findings are consistent with previous reports in which small-gauge vitrectomy was used.

Ding et al. reported the rate of the occurrence of POVH in the 23-gauge and 25-gauge groups as 4.3% and 5.2%, respectively (167 patients). The details on anticoagulation or antiplatelet medication were not mentioned. The PRP completion rate before vitrectomy was 17.1% and 15.5% in the 23-gauge and 25-gauge groups, similar to our data. The rates of pre-vitrectomy IV-anti VEGF agents administration (14.3%, 34.0%) and endodiathermy (17.1%, 7.2%) were lower than ours [32].

Schreur et al. reported POVH occurrence as 14% (including both 20-gauge and 23-gauge vitrectomy, 217 patients). However, the details on anticoagulation or antiplatelet medication were not mentioned. The prevalence of CKD (27%) and CAD (24%) was lower than that in our patient group. The percentage of patients with complete PRP (83%) was higher than that in our study, and the rate of previtrectomy IV anti-VEGF agent administration (17%) was lower than ours [33].

Ahn et al. reported POVH occurrence as 22.2% within one month after PPV (107 patients). The percentage of patients on anticoagulation therapy (38.9%) was higher than ours, the rate of IV-anti VEGF agent administration (17%) was lower than ours, and the percentage of patients with disk-involved FVM (47,2%) was similar to ours [34].

Takayama et al. reported a reoperation rate for POVH of 9.7% in small-gauge vitrectomy (452 patients). The percentages of patients on anticoagulation therapy (13.3%) and receiving pre-vitrectomy anti-VEGF (23.7%) and silicone oil tamponade (6.0%) were lower than ours, and the percentage of patients with complete PRP (68.1%) was higher than ours [17].

Khuthaila et al. reported a reoperation rate for POVH of 12.7% (173 patients). The percentage of patients with continuing anticoagulation medication (58.4%) was higher than ours, and the percentage receiving pre-vitrectomy anti-VEGF (6.9%) was lower than ours. No patients had silicone oil tamponade, and the details on TRD, FVM and intraoperative bleeding were not mentioned [14].

In the early era of 20-gauge vitrectomy, the influence of continuing anticoagulation or antiplatelet medicine was suggested to be related to POVH [12, 35, 36]. In recent studies of small-gauge diabetic vitrectomy, most evidence supports that the continued use of anticoagulation or antiplatelet medication in the peri-vitrectomy period does not increase the chance of POVH [13, 14, 17, 19, 32, 37]. Consistent with previous reports, we found that the occurrence of POVH was not significantly different between patients for whom anticoagulation or antiplatelet medications were continued (0/22) and patients for whom adjustments were made (11/198). One interesting finding of our work was that the long-term combined use of anticoagulation or antiplatelet medication in addition to aspirin was related to POVH, and patients taking combined anticoagulation or antiplatelet medications were more likely to develop POVH. The occurrence of POVH was significantly different in patients on dual antiplatelet (4,11) or dual antiplatelet combined with anticoagulation therapy (3,4) in our PDR patients.

Antiplatelet or anticoagulation agents were more commonly used in patients with persistent POVH than in patients without POVH. More patients with persistent POVH than patients without POVH were on dual antiplatelet therapy (4,11, p < 0.001) or a dual antiplatelet combined with anticoagulation therapy (3,4, p < 0.001).

Dual antiplatelet therapy is reported to have a positive linear relationship with the risk of postoperative bleeding in non-ocular surgery, especially in patients with anemia or poor renal function [5, 38]. Our findings on POVH supported that long-term use of antiplatelets could increase the risk ofpost-vitrectomy bleeding. We had three patients on dual antiplatelet therapy and three patients on dual antiplatelet therapy combined with anticoagulation therapy who had POVH, all of whom had CKD. We further showed that POVH developed after the restart of dual antiplatelet therapy.

Dual antiplatelet therapy combined with anticoagulation medication is used in patients with AF and deep vein thrombosis to prevent the development of stroke or recurrent atherothrombotic events and stent thrombosis and is a high-risk factor for postoperative bleeding. All patients in our study on combined anticoagulation and antiplatelet medications had end-stage renal failure. The previous consensus of general surgeons suggested that patients who undergo moderate- or high-risk bleeding procedures should maintain dual antiplatelet medications, bridge anticoagulation medication with LMWH, and restart anticoagulation medication as soon as possible [39]. We found that even with briding therapy, patients on combined antiplatelet and anticoagulation medications could develop POVH after restarting the medications.

Our data suggested that more attention should be given to managing intraoperative bleeding in PDR patients on long-term dual antiplatelet or antiplatelet therapy combined with anticoagulation medications. Therefore, follow-up in consideration of POVH should be recommended for PDR patients on these medications, especialy when antiplatelet or anticoagulation therapy is restarted.

CAD is commonly associated with PDR [40]. Interestingly, we showed that the presence of CAD, regardless of antiplatelet medications, was positively associated with POVH. We treated 53 patients unaware of their CAD and only 13 patients with diagnosed CAD who were on antiplatelet medications. We found that both conditions were associated with a higher occurrence of POVH. Therefore, our data suggested that the preoperative assessment for CAD is essential.

Previous non-ocular surgery data show that patients with end-stage renal failure are prone to developing postoperative bleeding [41]. In contrast, the data on vitrectomy in renal failure patients showed a contrary result: renal failure and hemodialysis, including heparin dialysis, do not appear to have a deteriorative influence on the outcomes of diabetic vitrectomy [18, 37, 42, 43]. Our results agree with the previous vitrectomy data; the occurrence of POVH was not different either between patients on dialysis (12) and patients not on dialysis (108) or between patients on heparin dialysis (5,7) and patients on non-heparin dialysis.

We investigated the known risk factors for POVH, including neovascular on the neovascularization of the disk [13], the completion of PRP [14], the presence of TRD [17], residual FVM or neovascularization of the membrane on the disk, residual vitreous cortex, and the ingrowth of the fibrovascular membrane into the incision [21, 22], in our group of PDR patients. Pre-PPV IVR, endodiathermy, intraoperative photocoagulation, peripheral vitrectomy with scleral depression, TA-assisted vitrectomy, and silicone oil tamponade, which are thought to reduce the risk of POVH, were used as required [15]^18^. Unexpectedly, we demonstrated no correlation between the extent of FVM or surgical maneuvers and the occurrence of POVH. We had a high prevalence of patients with disk-involved FVPs (44.5%) and incomplete PRP (82.3%), indicating complicated PDR. The corresponding complicated vitrectomy procedures resulted in a high prevalence of patients who had retinectomy (29.5%), severe intraoperative bleeding requiring endodiathermy (40.0%), and silicone oil tamponade (33.6%). Our vitrectomy techniques may reduce the risk of POVH in complicated PDR cases. Even with those techniques, POVH in patients with complicated PDR was unavoidable. Our results suggested that POVH should still be considered even after all vitrectomy techniques to reduce the risk of POVH have been applied in patients with severe complicated PDR.

IV-anti-VEGF agents are an effective method to lessen the risk of POVH [15, 22, 44]. We had a high percentage of patients who received pre-PPV IVR (86.4%). The role of pre-PPV IVR on POVH required further investigations with more patients without pre-PPV IVR.

## Limitations

This was a retrospective cohort study, and the role of POVH-related factors needs further exploration in a prospective cohort. We did not analyze patients with spontaneously resolved POVH when we investigated the factors related to POVH. Previous work has shown that postoperative IV-anti-VEGF agent administration effectively resolves VH [21, 45]. We did not include the details on the management of POVH before the re-PPV. We failed to obtain postoperative VA data in this cohort.

## Conclusions

This study aimed to investigate the relationship between anticoagulation, antiplatelet medication, and POVH in a group of complicated PDR patients with a high prevalence of systemic complications who underwent small-gauge vitrectomy. We identified long-term use of anticoagulation or antiplatelet medication, the presence of CAD, and younger age as three independent factors related to POVH. The extent of FVM, use of retinectomy, or silicone oil did not affect the occurrence of POVH. In PDR patients on long-term antiplatelet or anticoagulation medication, particular attention should be given to controlling intraoperative bleeding, and follow-up for POVH should be scheduled.

## Data Availability

The dataset(s) supporting the conclusions of this article is(are) available in zhao, meng (2022), “” PDR patients and vitrectomy, Mendeley Data, V1, doi: 10.17632/gdkpjnjv45.1.

## List of abbreviations

AIC: Akaike information criterion
CAD: Coronary artery disease
CKD: Chronic kidney diseases
CVD: Cardio-cerebral vascular diseases
FVM: Fibrovascular proliferative membrane
HTN: Hypertension
IV: Intravitreal
IVR: IV ranibizumab
LMWH: Low-molecular-weight heparin
PDR: Proliferative diabetic retinopathy
POVH: Post vitrectomy vitreous hemorrhage
PPV: Pars plana vitrectomy
PRP: Pan retinal photocoagulation
ROC: Receiver operating characteristic curve curve
TRD: Tractional retinal detachment
VA: Visual acuity
VEGF: Vascular endothelial growth factor
VH: Vitreous hemorrhage
